# Gyrotactic micro-organism flow of Maxwell nanofluid between two parallel plates

**DOI:** 10.1038/s41598-021-94543-4

**Published:** 2021-07-26

**Authors:** Yun-Jie Xu, Muhammad Bilal, Qasem Al-Mdallal, Muhammad Altaf Khan, Taseer Muhammad

**Affiliations:** 1 School of Engineering, Huzhou University, Huzhou, 313000 People’s Republic of China; 2 Department of Mathematics, City University of Science and Information Technology, Peshawar, Pakistan; 3 Department of Mathematical Sciences, UAE University, P. O. Box 15551, Al Ain, United Arab Emirates; 4 Institute for Groundwater Studies, Faculty of Natural and Agricultural Sciences, University of Free State, Bloemfontein, South Africa; 5 Department of Mathematics, College of Sciences, King Khalid University, Abha, 61413 Saudi Arabia

**Keywords:** Engineering, Mathematics and computing, Nanoscience and technology

## Abstract

The present study explores incompressible, steady power law nanoliquid comprising gyrotactic microorganisms flow across parallel plates with energy transfer. In which only one plate is moving concerning another at a time. Nonlinear partial differential equations have been used to model the problem. Using Liao's transformation, the framework of PDEs is simplified to a system of Ordinary Differential Equations (ODEs). The problem is numerically solved using the parametric continuation method (PCM). The obtained results are compared to the boundary value solver (bvp4c) method for validity reasons. It has been observed that both the results are in best settlement with each other. The temperature, velocity, concentration and microorganism profile trend versus several physical constraints are presented graphically and briefly discussed. The velocity profile shows positive response versus the rising values of buoyancy convection parameters. While the velocity reduces with the increasing effect of magnetic field, because magnetic impact generates Lorentz force, which reduces the fluid velocity.

## Introduction

The association of a conducting fluid with a magnetic field is a well-known area of Magnetohydrodynamics (MHD). Fluid flow across parallel plates has many research and production applications; such a fluid flow helps to pinch flow, which is very useful in bearings for lubrication purposes in metal, cooling towers and food production, hydrodynamic devices, petrochemical industry, fog forming and scattering, polymer processing, and protecting crops from freezing^1^. Squeezing flow is useful for lubrication as that protects lubricants from losing viscosity unexpectedly at elevated temperatures or in such harsh working conditions. Stefan^2^ was the first to investigate gripping flow in a device for lubrication purposes. Hakeem et al.^3^ conducted an analytical study on entropy production for viscoelastic fluid flow including an angled magnetic field and non-linear thermal radiation characteristics with a heat source and sink across a stretched sheet. Awan et al.^4^ has studied the applications of cumulative magnetic and electric field in nanoliquid moving through two parallel plates. They employed RK4 and Adams techniques for the stability, accuracy and solution of the problem. Shafiq et al.^5^ used RSM to evaluate the incremental influence of thermal radiation on energy transference optimization, which correlates to Darcy–Forchheimer (DF) carbon nanotube flow along a stretched spinning surface. The role of Casson carbon nanotubes in boundary layer flow is being studied, having implications for both single-walled and multi-walled CNTs. The macroscopic flow and microscopic properties and information of polymeric fluid between parallel two plates using modified multiscale technique are scrutinized by Yan et al.^6^. Biswal et al.^7^ reported the copper and silver nanofluid flow in a permeable channel with the consequences of magnetic field using Brinkman and Garnett-Maxwell models for viscosity and strong electric conductivity. Ganga et al.^8^ explored the role of internal heat generation/absorption on steady radiative MHD boundary-layer flow of a viscous, incompressible nanofluid across a vertical plate using RK4 procedure. The nanomaterial volume fraction profile reduces the action of heat generation and increases in the presence of heat absorption. Ganga et al.^9,10^ evaluated the consequences of internal heat production and absorption, as well as viscous and ohmic dissipations, on the two-dimensional radiative MHD boundary-layer flow of a viscous nanofluid across a vertical plate.

The heat and mass transport concept has a wide range of applications. It's essential in engineering, industry, semiconductors, and solar devices, as well as bio separations and metallurgy. The main fields where mass transfer phenomena exist are biomedical, pharmacokinetics research, and medication metabolism in the body, tissue engineering, including the construction of artificial organs, oxygen transport in the lungs and dialysis systems, and catalytic converter in automobiles. References^11,12^ contains many studies that emphasize the importance of mass transfer convective flow. Ahmadian et al.^13,14^ numerically computed the unsteady *Ag-MgO* (silver magnesium) hybrid nano liquid flow due to fluctuating of rotating disk with heat and mass transmission characteristics. The wavy non-flat surface enhances the heat transport rate up to 15%, then normal flat surfaces. Bilal et al.^15^ reported the heat and mass transmission through hybrid nanofluid flow across a stretching cylinder using carbon and ferric oxide nanoparticles. The results indicate that the proposed Nano composites are the most effective means of improving heat exchange and can also be used for refrigeration system. Shuaib et al.^16^ elaborated the ions transmission by considering Nernst-Plank’s equation along with Navier Stoke’s and developed a numerical model for the simulation of ionic species. Acharya et al.^17–19^ demonstrated the boundary layer Cattaneo-Christov model of mass and heat transmission of an upper-convected Maxwell nanofluid passing through an inclined extending substrate in the presence of a magnetic force in a systematic way.

Microorganisms are single-celled organisms; they live everywhere, as in rodents, humans, and plant bodies. They're so much thicker than water, due to which, microorganisms become a cause of bio convection. The bio convection phenomenon is presented by oxytocic bacteria that swim microorganisms up. The bio-convection model addresses directed swimming cells that are related to the organisms of the microorganism. Bio convection's physical importance is efficiently assorted in biofuels, ethanol, and a variety of industrial and environmental structures. Aside from that, bio convection of nanoparticles is linked to stratification density and pattern forming, this occurs as a result of the interaction of microorganisms, buoyancy forces, and nanomaterials. When gyrotactic microorganisms are present, the suspension stability of nanoparticles is often found to be significantly improved. Many researchers have written on these fascinating phenomena in recent years. The functionality of bio convection is established by an increase in the concentrations of motile microorganisms. Geng and Kuzenstov's partnership^20^ looks at the interaction between nanomaterial and microbes. The addition of gyrotactic microorganisms improves the Nano composites' stability, according to the researchers. Zuhra et al.^21^ explore the interaction of two parallel plates comprising nanomaterials and gyrotactic microorganisms in a time-dependent second-grade fluid. They discovered that as the unsteadiness effect is improved, fluid velocity raises, while it is reduces with the viscoelastic term effect. The viscous nanoliquid flow over a stretching layer containing gyrotactic microorganisms was studied by Rehman et al.^22^. They discovered that increasing the values of Peclet numbers lowers the density of motile microorganisms. Khan et al.^23^ inspected the bioconvection characteristics of nano-size particles in presence of chemical reaction and Lorentz force in non-Newtonian fluid over moving surface. Three dimensional nanoliquid flow passes via parallel plates with the effects of electric field and heat transfer characteristics using Matlab bvp4c and RK4 is analyzed by Shuaib et al.^24^. Acharya et al.^25,26^ explored the bioconvection water-based nanofluid flow across a permeable surface in the presence of gyrotactic microorganisms. The flow study has been studied with the influence of the surface slip condition.

The magnetic force in the field of hydrodynamics has several applications, especially the electro conducting fluid squeezing flow between two parallel plates have many applications. Which has been already explained in the above paragraphs, keep in view these uses of conducting squeezed flow; we extend the idea of Ferdows et al.^27^. Within the established mathematical model, incompressible squeezing flow of viscous fluid with heat transfer under magnetic field has been studied. The next section consists of formulation and discussion related to the problem.

## Mathematical formulation

**Figure 1:**
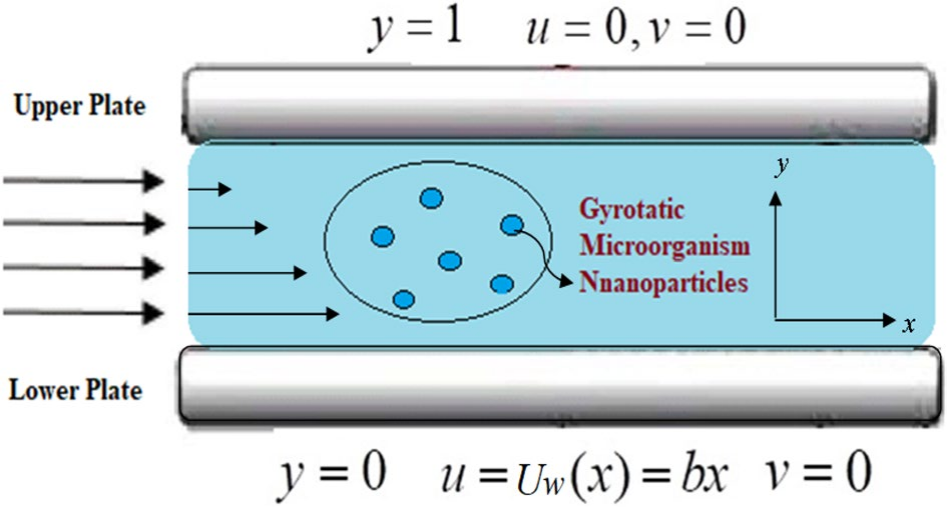
Fluid flow between two parallel plates.

We consider incompressible, steady power law nanoliquid comprising gyrotactic microorganisms flow across parallel plates. The lower plate is at rest in this case. *U*_*w*_ is the uniform velocity of the upper layer. The gap $h^{ * }$ between the plates distinguishes them. Figure 1 illustrates the flow geometry. Both the horizontal *y*-axis and the vertical *x*-axis are subjected to a variable magnetic field. The lower plate is kept at temperature $T_{0}$, while the upper plate is kept at a constant temperature $T_{\infty }$. The Lorentz force $\mathop{J}\limits^{\rightharpoonup} \times \mathop{B}\limits^{\rightharpoonup}$ is used to modify the equations for non-conducting plates. The continuity equation, electricity, and Maxwell can all be written as^27^ under the above assumptions:

1 $$ \frac{\partial u}{{\partial x}} + \frac{\partial v}{{\partial y}} = 0, $$

2 $$ u\frac{\partial u}{{\partial x}} + v\frac{\partial u}{{\partial y}} = \frac{1}{\rho }\left[ { - \mu \frac{\partial }{\partial y}\left( { - \frac{\partial u}{{\partial y}}} \right)^{n} - \sigma_{e} B_{0}^{2} u + g\rho \beta \left( {T - T_{\infty } } \right) + g\rho \beta \left( {C - C_{\infty } } \right) + g\rho \beta \left( {n - n_{\infty } } \right)} \right], $$

3 $$ u\frac{\partial T}{{\partial x}} + v\frac{\partial T}{{\partial y}} - \alpha \left( {\frac{{\partial^{2} T}}{{\partial y^{2} }}} \right) - \tau \left( {\frac{{D_{T} }}{{T_{\infty } }}\left( {\frac{\partial T}{{\partial y}}} \right)\left( {\frac{\partial T}{{\partial y}}} \right) + D_{B} \frac{\partial T}{{\partial y}}\frac{\partial C}{{\partial y}}} \right) = 0, $$

4 $$ u\frac{\partial C}{{\partial x}} + v\frac{\partial C}{{\partial y}} - D_{B} \left( {\frac{{\partial^{2} C}}{{\partial y^{2} }}} \right) - \frac{{D_{T} }}{{T_{\infty } }}\left( {\frac{{\partial^{2} T}}{{\partial y^{2} }}} \right) = 0, $$

5 $$ u\frac{\partial n}{{\partial x}} + v\frac{\partial n}{{\partial y}} - D_{n} \left( {\frac{{\partial^{2} n}}{{\partial y^{2} }}} \right) + \frac{{dW_{c} }}{{C_{w} - C_{\infty } }}\frac{\partial }{\partial y}\left( {n\frac{\partial C}{{\partial y}}} \right) = 0, $$

6 $$ u\frac{{\partial B_{y} }}{\partial x} + B_{y} \frac{\partial u}{{\partial x}} = v\frac{{\partial B_{x} }}{\partial x} + B_{x} \frac{\partial v}{{\partial x}} + \frac{1}{{\delta \mu_{2} }} \left(\frac{{\partial^{2} B_{y} }}{{\partial x^{2} }} + \frac{{\partial^{2} B_{y} }}{{\partial y^{2} }} \right), $$

The imposed boundary conditions for infinite rotating disk are as follows:

7 $$ \begin{gathered} u = U_{w} (x) = b_{x} ,\,\,\,\,v = 0,\,\,\,T = T_{w} ,\,\,\,C = C_{w} ,\,\,\,n = n_{w} ,\,\,\,Bx{ = 0, }By = M_{0} \,\,\,\,\,{\text{at}}\,\,y = 0, \hfill \\ {\text{As}}\,\,y \to \infty ,\,\,u \to 0,\,\,T \to T_{\infty } ,\,\,C \to C_{\infty } ,\,\,n \to n_{\infty } ,\,\,\,Bx{ = 0, }\,By = 0\,\,\,\,{\text{at}}\,\,y = 1,\,\,\, \hfill \\ \end{gathered} $$

Here, g, $T_{\infty }$, $T$, $\beta$, $D_{T}$, $D_{B}$, $\sigma_{e}$, $B_{0}$ and $C_{w}$ is the acceleration, free stream temperature, fluid temperature, heat source/sink parameter, thermophoresis coefficient, Brownian diffusion coefficient, electrical conductivity, magnetic field strength and the concentration.

Where, $c_{p}$, C, $\tau$, $D_{n}$, $W_{c}$, *K*, $\rho$ and $\mu_{2}$ is the specific heat, concentration, effective heat capacitance, diffusivity of the microorganisms, cell swimming speed, thermal diffusion ratio, density and magnetic permeability of the fluid respectively.

The structure of PDEs will be reduced into a function of single variable, using the following conversion^27^:

8 $$ \begin{gathered} \eta = \frac{y}{x}{\text{Re}}_{x}^{{\frac{1}{(n + 1)}}} ,\,\,\,\,\,\,\psi = xU_{w} {\text{Re}}_{x}^{{\frac{1}{(n + 1)}}} f(\eta ),\,\,\,\,\,\,{\text{Re}}_{x} \, = \frac{{x^{n} (U_{w} )^{{\left( {2 - n} \right)}} }}{uf},\,\,\,\chi (\eta ) = \frac{{n - n_{\infty } }}{{n_{w} - n_{\infty } }},\,\,\,\,\,\theta (\eta ) = \frac{{T - T_{\infty } }}{{T_{w} - T_{\infty } }},\, \hfill \\ \varphi (\eta ) = \frac{{C - C_{\infty } }}{{C_{w} - C_{\infty } }},\,\,B_{x} = \frac{{\alpha xM_{0} }}{2h}h(\eta ). \hfill \\ \end{gathered} $$

The following system of ODE is formed by using Eq. (8) in Eqs. (1)–(7)

9 $$ n( - f^{\prime\prime})^{n - 1} f^{\prime \prime \prime} + \frac{2n}{{n + 1}}ff^{\prime\prime} - f^{{\prime}{2}} - Mf^{\prime} + \lambda_{1} \theta + \lambda_{2} \varphi + \lambda_{3} \chi = 0, $$

10 $$ \theta^{\prime\prime} + Nb\Pr \theta^{\prime}\varphi ^{\prime} + \frac{2n}{{n + 1}}Prmf\theta ^{\prime} + \Pr Nt\theta ^{{\prime}{2}} = 0, $$

11 $$ \varphi^{\prime\prime} + \frac{2n}{{n + 1}}Prm\,Lef\varphi^{\prime} + \frac{Nt}{{Nb}}\theta^{\prime\prime} = 0, $$

12 $$ \chi^{\prime\prime} + \frac{2n}{{n + 1}}PrmLbf\chi^{\prime} - Pe(\chi^{\prime}\varphi^{\prime} + \chi \varphi^{\prime\prime}) = 0, $$

13 $$ h^{\prime\prime} + R_{2} Btfh^{\prime} - 2R_{2} Bthf^{\prime} + \frac{{2R_{2}^{2} Bt}}{{R_{1} }}f = 0. $$

The transform boundary conditions are spelled out as follows:

14 $$ \begin{gathered} \eta = 0,\,\,\,\,f = 0,\,\,\,f^{\prime} = 1,\,\,\,\,\theta = 1,\,\,\,\varphi = 1,\,\,\chi = 1,\,\,\,\,h = 1. \hfill \\ \eta \to \infty ,\,\,f \to 0,\,\,f^{\prime} \to 0,\,\,\,\theta \to 0,\,\,\,\varphi \to 0,\,\,\,\,\chi \to 0,\,\,\,\,h \to 0. \hfill \\ \end{gathered} $$

here, $R_{1}$ = $\frac{{h^{2} }}{\nu }$ and $R_{2}$ = $\frac{{\alpha h^{2} }}{2\nu }$ is the Reynolds number based on the speed of the plates. where, the magnetic strength, modified Prandtl number, Prandtl number, Peclet number, Brownian motion, Thermophoresis parameter, Lewis number, Bioconvection Lewis number, Buoyancy convection coefficient due to temperature, Buoyancy convection coefficient due to concentration and Batchlor number are defined as^27,28^:

15 $$ \begin{gathered} M = \left( {\sigma B_{0}^{2} /b\rho } \right),\,\,Prm = \frac{{bx^{2} }}{\alpha },\,\,\Pr = \nu /\alpha ,\,\,{\text{P}} e = \left( {dwc/D_{n} } \right),\,\,Nb = \left( {\left( {\rho c_{p} } \right)D_{B} \left( {C_{w} - C_{\infty } } \right)} \right)/\left( {\nu T_{\infty } \left( {\rho c_{f} } \right)} \right),\,\,Bt = \sigma \mu_{2} \nu , \hfill \\ Nt = \left( {\left( {\rho c_{p} } \right)D_{T} \left( {T_{w} - T_{\infty } } \right)} \right)/\left( {\nu T_{\infty } \left( {\rho c_{f} } \right)} \right),\,\,\,Le = \nu /D_{B} ,\,\,\,Lb = \alpha /D_{n} ,\,\,\,\lambda_{1} = Gr_{x} /{\text{Re}}_{x} ,\,\,\,\lambda_{2} = Gr_{c} /{\text{Re}}_{x} ,\,\,\lambda_{3} = Gr_{n} /{\text{Re}}_{x} . \hfill \\ \end{gathered} $$

The skin friction $C_{f}$, heat transmission rate $Nu_{x}$, mass transmission rate $Sh_{x}$ and density of motile micro-organism $Nn_{x}$ are given as:

16 $$ C_{f} = 2\tau_{w} /\rho U_{w}^{2} ,\,\,\,\,Sh_{x} = xq_{w} /k\Delta T,\,\,\,\,Nu_{x} = xq_{m} /D_{B} \Delta C,\,\,\,\,Nn_{x} = xq_{n} /D_{n} \Delta n. $$

where, $q_{w} = - k\left( {\partial T/\partial y} \right)_{y = o}$, $\tau_{w} = - \mu \left( {\partial u/\partial y} \right)_{y = o}$, $q_{n} = - D_{n} \left( {\partial n/\partial y} \right)_{y = o}$ and $q_{m} = - D_{B} \left( {\partial C/\partial y} \right)_{y = o}$ is the heat flux, shear stress, flux of motile microorganism and mass flux at plate surface at the surface of the plate.

Now using above terminologies, Eq. (16) yields:

$$ \left. \begin{gathered} C_{f} {\text{Re}}_{x}^{{1/\left( {n + 1} \right)}} = 2\left| {f^{\prime\prime}\left( 0 \right)} \right|^{n - 1} f^{\prime\prime}\left( 0 \right),\,\,\,{\text{Re}}_{x}^{{ - 1/\left( {n = + 1} \right)}} Nu_{x} = - \theta^{\prime}\left( 0 \right), \hfill \\ {\text{Re}}_{x}^{{ - 1/\left( {n = + 1} \right)}} Sh_{x} = - \varphi^{\prime}\left( 0 \right),\,\,\,{\text{Re}}_{x}^{{ - 1/\left( {n = + 1} \right)}} Nn_{x} = - \chi^{\prime}\left( 0 \right). \hfill \\ \end{gathered} \right\} $$

## Numerical solution

The following steps present the fundamental concept of applying the PCM method to an ODE system (9–13) with a boundary condition (14).

Step 1: The BVP system is being converted into a first-order system of ODE
  The following functions will be introduced:
  18 $$ \left. \begin{gathered} \zeta_{1} (\eta ) = f(\eta ),\,\,\,\,\zeta_{2} (\eta ) = f^{\prime}(\eta ),\,\,\zeta_{3} (\eta ) = f^{\prime\prime}(\eta ),\,\,\,\zeta_{4} (\eta ) = \theta (\eta ),\,\,\zeta_{5} (\eta ) = \theta^{\prime}(\eta ),\,\, \hfill \\ \zeta_{6} (\eta ) = \varphi (\eta ),\,\,\zeta_{7} (\eta ) = \varphi^{\prime}(\eta ),\,\,\zeta_{8} (\eta ) = \,\,\chi (\eta ),\,\,\zeta_{9} (\eta ) = \chi^{\prime}(\eta ),\,\,\zeta_{10} (\eta ) = h(\eta ),\,\,\zeta_{11} (\eta ) = h^{\prime}(\eta ). \hfill \\ \end{gathered} \right\} $$
  Using transformations (18) into the BVP (9–13) and (14), to get:
  19 $$ n( - \zeta_{3} (\eta ))^{n - 1} \zeta_{3}^{\prime } (\eta ) + \frac{{b^{2} x}}{n + 1}\zeta_{3} (\eta ) - M\zeta_{2} (\eta ) + \lambda_{1} \zeta_{4} (\eta ) + \lambda_{2} \zeta_{6} (\eta ) + \lambda_{3} \zeta_{8} (\eta ) = 0, $$
  20 $$ \zeta^{\prime}_{5} (\eta ) + Nb\Pr \zeta_{5} (\eta )\zeta_{7} (\eta ) + \frac{2n}{{n + 1}}\Pr \zeta_{1} (\eta )\zeta_{5} (\eta ) + PrmNt\zeta_{5}^{2} (\eta ) = 0, $$
  21 $$ \zeta_{7}^{\prime } (\eta ) + \frac{2n}{{n + 1}}Le\,Prm\zeta_{1} (\eta )\zeta_{5} (\eta ) + \frac{Nt}{{Nb}}\zeta_{5}^{\prime } (\eta ) = 0, $$
  22 $$ \zeta_{9}^{\prime } (\eta ) + \frac{2n}{{n + 1}}PrmLb\zeta_{1} (\eta )\zeta_{9} (\eta ) - Pe(\zeta_{9} (\eta )\zeta_{7} + \chi \zeta_{7}^{\prime } (\eta )) = 0, $$
  23 $$ \zeta_{11}^{\prime } (\eta ) + R_{2} Bt\zeta_{1} (\eta )\,\zeta_{11} (\eta ) - 2R_{2} Bt\zeta_{10} (\eta )\zeta_{2} (\eta ) + \frac{{2R_{2}^{2} Bt}}{{R_{1} }}\zeta_{1} (\eta ) = 0. $$
  The associated boundary conditions are:
  24 $$ \begin{gathered} \eta = 0,\,\,\,\,\zeta_{1} (\eta ) = 0,\,\,\,\zeta_{2} (\eta ) = 1,\,\,\,\,\zeta_{4} (\eta ) = 1,\,\,\,\zeta_{6} (\eta ) = 0,\,\,\zeta_{8} (\eta ) = 1,\,\,\,\,\zeta_{10} (\eta ) = 1, \hfill \\ \eta \to \infty ,\,\,\,\,\zeta_{2} (\eta ) \to 0,\,\,\,\,\zeta_{4} (\eta ) \to 0,\,\,\,\,\zeta_{6} (\eta ) \to 0,\,\,\,\,\zeta_{8} (\eta ) \to 0,\,\,\,\,\zeta_{10} (\eta ) \to 0. \hfill \\ \end{gathered} $$
Step 2: The embedding term p is introduced as:
  We will thoroughly add the continuation parameter p in the system (19–23) to obtain an ODE system in a p-parametric family.
  25 $$ n(( - \zeta_{3} (\eta ) - 1)p)^{n - 1} \zeta_{3}^{\prime } (\eta ) + \frac{{b^{2} x}}{n + 1}\zeta_{3} (\eta ) - M\zeta_{2} (\eta ) + \lambda_{1} \zeta_{4} (\eta ) + \lambda_{2} \zeta_{6} (\eta ) + \lambda_{3} \zeta_{8} (\eta ) = 0, $$
  26 $$ \zeta^{\prime}_{5} (\eta ) + (Nb\Pr \zeta_{7} (\eta ) + \frac{2n}{{n + 1}}\Pr \zeta_{1} (\eta ))(\zeta_{5} (\eta ) - 1)p + Prm\,Nt\zeta_{5}^{2} (\eta ) = 0, $$
  27 $$ \zeta_{7}^{\prime } (\eta ) + \frac{2n}{{n + 1}}Prm\,Le\zeta_{1} (\eta )\zeta_{5} (\eta ) - \zeta_{7} (\eta ) + (\zeta_{7} (\eta ) - 1)p + \frac{Nt}{{Nb}}\zeta_{5}^{\prime } (\eta ) = 0, $$
  28 $$ \zeta_{9}^{\prime } (\eta ) + \left (\frac{2n}{{n + 1}}PrmLb\zeta_{1} (\eta )\, - Pe\zeta_{7} (\eta )\right)(\zeta_{9} (\eta ) - 1)p - Pe\chi \zeta_{7}^{\prime } (\eta ) = 0, $$
  29 $$ \zeta_{11}^{\prime } (\eta ) + R_{2} Bt\zeta_{1} (\eta )(\zeta_{11} (\eta ) - 1)p - 2R_{2} Bt\zeta_{10} (\eta )\zeta_{2} (\eta ) + \frac{{2R_{2}^{2} Bt}}{{R_{1} }}\zeta_{1} (\eta ) = 0. $$
Step 3: Differentiating by parameter ‘p’
  After differentiating Eqs. (25–29) with respect to parameter p, you will get the following system in terms of parameter p sensitivities.
  30 $$ V^{\prime} = AV + R, $$
  where A denotes the coefficient matrix, R denotes the remainder.
  31 $$ \frac{{d\zeta_{i} }}{d\tau }, $$
  where *i* = 1, 2, …11*.*
Step 4: For each element, assert the principle of superposition and define the Cauchy problem.
  32 $$ V = aU + W, $$
  where U, W, and a denote unknown vector functions and blend coefficients, respectively. For each component, solve the two Cauchy problems listed below.
  33 $$ U = aU, $$
  34 $$ W^{\prime} = AW + R, $$
  We get the approximate solution Eq. (32) by plugging it into the original Eq. (30).
  35 $$ (aU + W)^{\prime} = A(aU + W) + R, $$
Step 5: Solving the Cauchy problems
  This work employs a numerical implicit scheme, which is depicted below.
  36 $$ \frac{{U^{i + 1} - U^{i} }}{\Delta \eta } = AU^{i + 1} ,\,\,or\,\,\,\,(I - \Delta \eta A)U^{i + 1} = U^{i} , $$
  37 $$ \frac{{W^{i + 1} - W^{i} }}{\Delta \eta } = AW^{i + 1} ,\,\,or\,\,\,\,(I - \Delta \eta A)W^{i + 1} = W^{i} , $$
  where the iterative form of the solution is obtained
  38 $$ U^{i + 1} = (I - \Delta \eta A)^{ - 1} U^{i} , $$
  39 $$ W^{i + 1} = (I - \Delta \eta A)^{ - 1} (W^{i} + \Delta \eta R). $$

## Results and discussion

### Velocity profile

**Figure 2:**
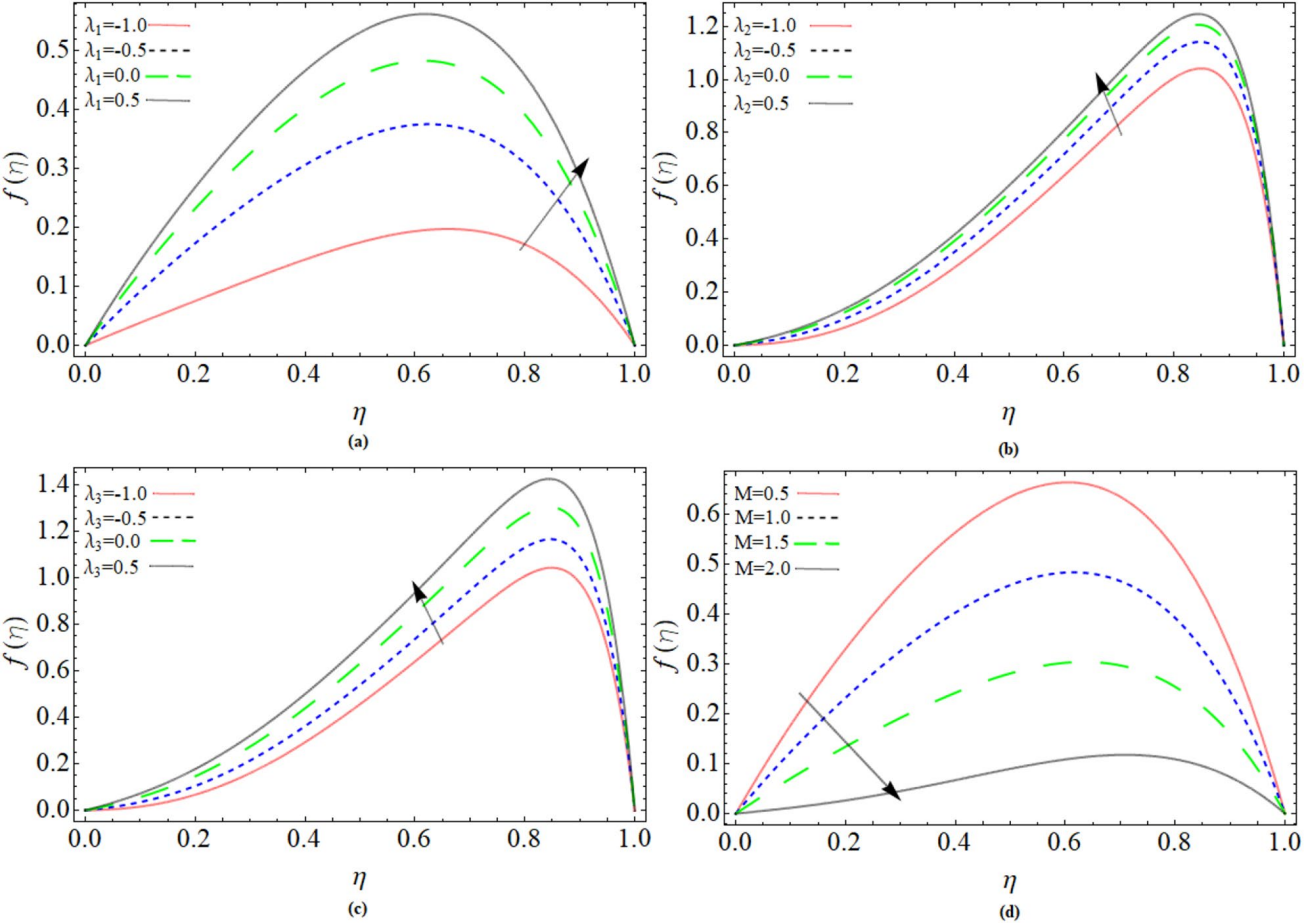
Buoyancy convection term due to temperature $\lambda_{1}$, concentration $\lambda_{2}$ and microorganism $\lambda_{3}$ and magnetic parameter* M* effect on velocity profile $f\left( \eta \right)$.

Buoyancy convection parameter due to temperature $\lambda_{1}$, concentration $\lambda_{2}$ and microorganism $\lambda_{3}$ and magnetic parameter* M* influence on velocity profile $f\left( \eta \right)$ has been illustrated via Fig. 2a–d. A downward direction flow is represented by a $\lambda_{1}$ value less than zero, while a vertical upward flow is represented by a $\lambda_{1}$ value greater than zero. Figure 2a–c display that the velocity profile shows positive response versus the rising values of Buoyancy convection parameters. On other hand, velocity is reduces with increasing effect of magnetic field, because magnetic impact generated Lorentz force, which reduces fluid velocity $f\left( \eta \right)$.

### Temperature profile

**Figure 3:**
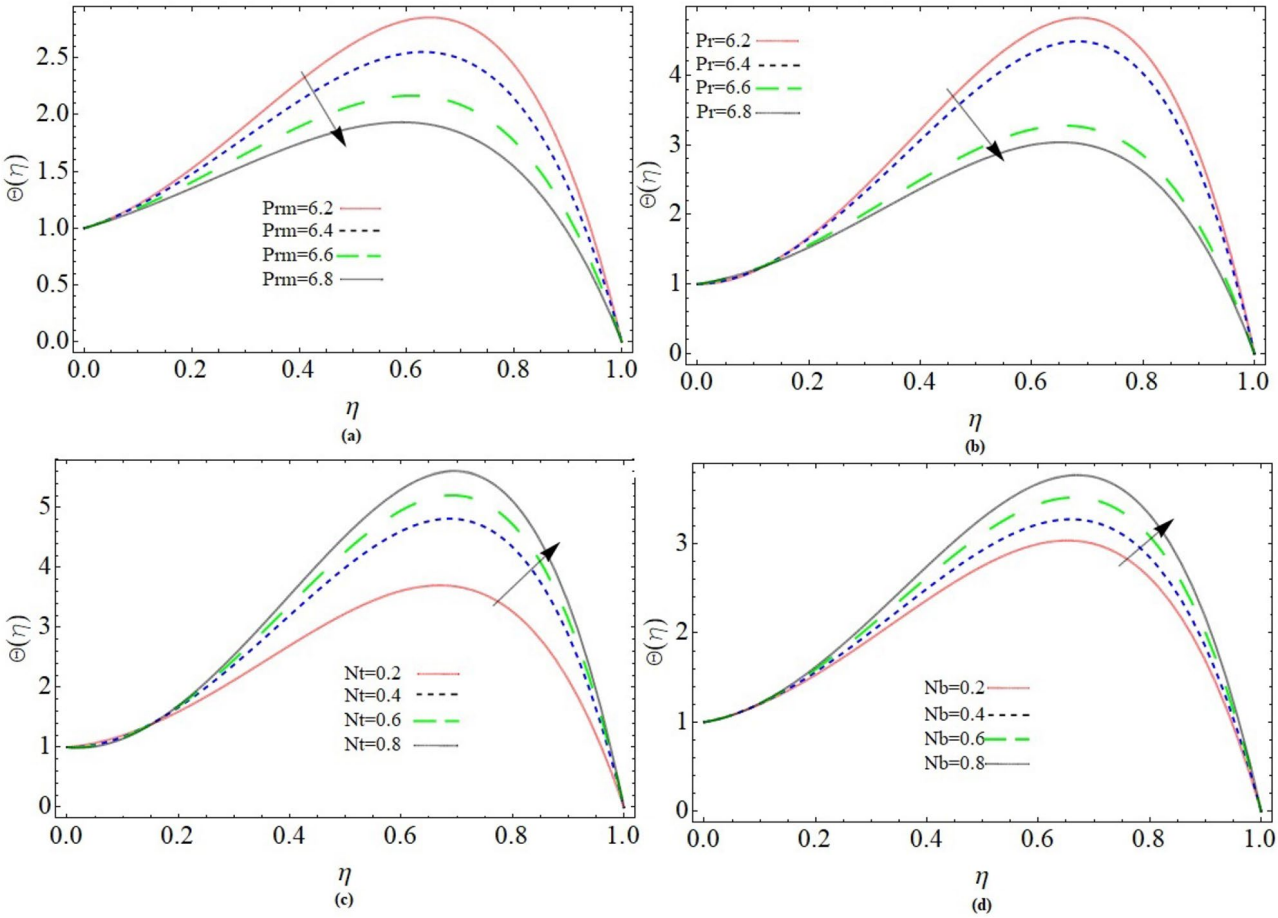
Parameter *Prm*, *Pr, Nb* and *Nt* effect on temperature profile $\Theta \left( \eta \right)$.

Magnetic Prandtl number *Prm*, *Nb*, *Nt* and Prandtl number *Pr* effect on temperature profile $\Theta \left( \eta \right)$ has been presented through Fig. 3a–d. The rising effect of magnetic Prandtl number and Prandtl number significantly reduces the fluid temperature Fig. 3a,b respectively. Actually, the kinematic viscosity of fluid increases, and thermal diffusivity decreases with rising values of Prandtl number, that’s why such trend has been observed. Brownian behavior is considered as the non-movement of fluid molecules over the plate's surface. Brownian motion induces heat by increasing the unspecific motion of liquid particles. As a result, the liquid temperature rises, as does the thickness of the thermal boundary layer as shown in Fig. 3c. Furthermore, as the thermophoresis component improves the smallest nanomaterials are escorted away from heated surface and toward the cold surface. As a consequence, as shown in Fig. 3d, a larger number of small nanoparticles are drawn away from the warm surface, increasing the liquid temperature.

### Concentration profile

**Figure 4:**
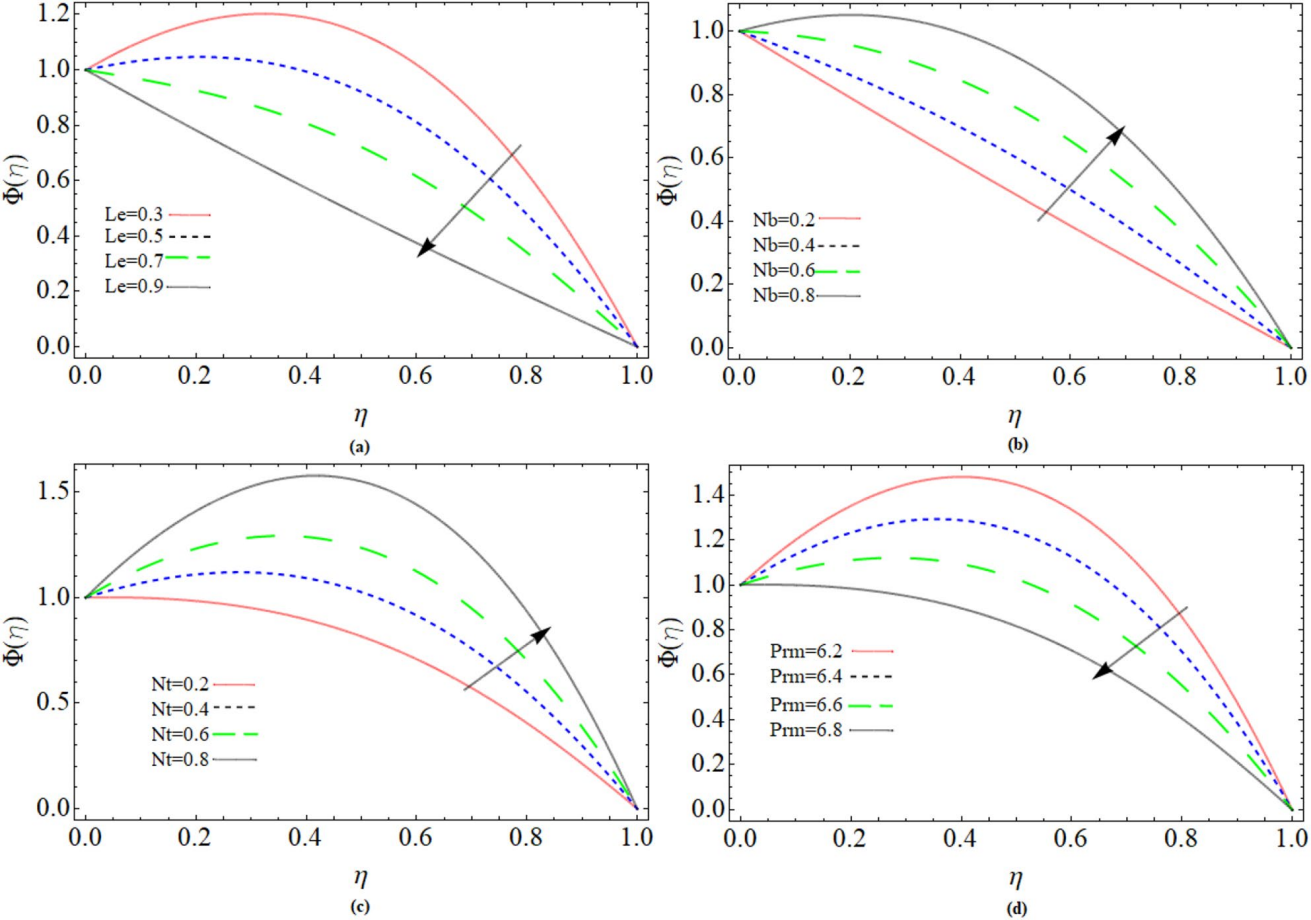
Parameter *Le*, *Nt*, *Nb* and *Prm* effect on concentration profile $\Phi \left( \eta \right)$.

Lewis number *Le*, Thermophoresis parameter *Nt*, Brownian motion parameter *Nb* and magnetic Prandtl number *Prm* effect on concentration profile $\Phi \left( \eta \right)$ has been elaborated through Fig. 4a–d. The concentration distribution declines with rising values of Lewis number *Le*. Because the rate of molecular diffusion reduces and kinematic viscosity enhances with the effect of Lewis number shown in Fig. 4a. The mass transmission rate strengthens with growing values of Brownian motion Fig. 4b. Similar behavior of mass transport has been observed versus Thermophoresis parameter in Fig. 4c. While transmission rate of mass distribution is negatively affected by magnetic Prandtl number displays in Fig. 4d.

### Magnetic strength profile

**Figure 5:**
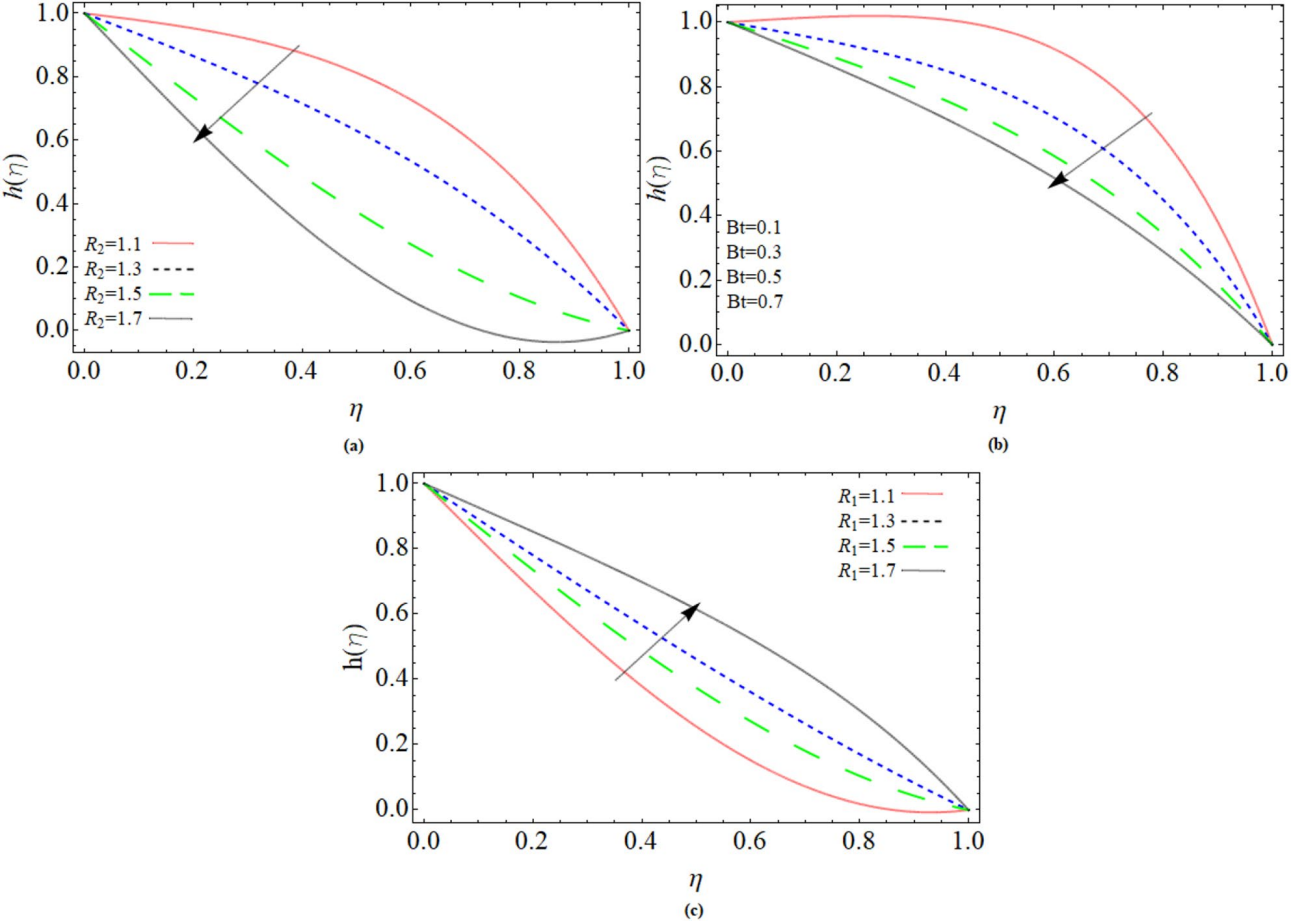
Parameter R_1_, R_2_ and *Bt* effect on magnetic strength profile $h\left( \eta \right)$.

Magnetic Reynolds number R_1_, R_2_ and Batchlor number *Bt* effect on magnetic strength profile $h\left( \eta \right)$ has been displayed via Fig. 5a–c. The magnetic strength profile reduces with increment in magnetic Reynold number and Batchlor number *Bt* respectively. Physically, the improvement of magnetic Reynold number and Batchlor number *Bt* reduces the magnetic diffusivity and enhances the kinematic viscosity of fluid flow; as a result, magnetic strength profile reduces as presented in Fig. 5a,b respectively.

### Gyrotactic microorganism profile

**Figure 6:**
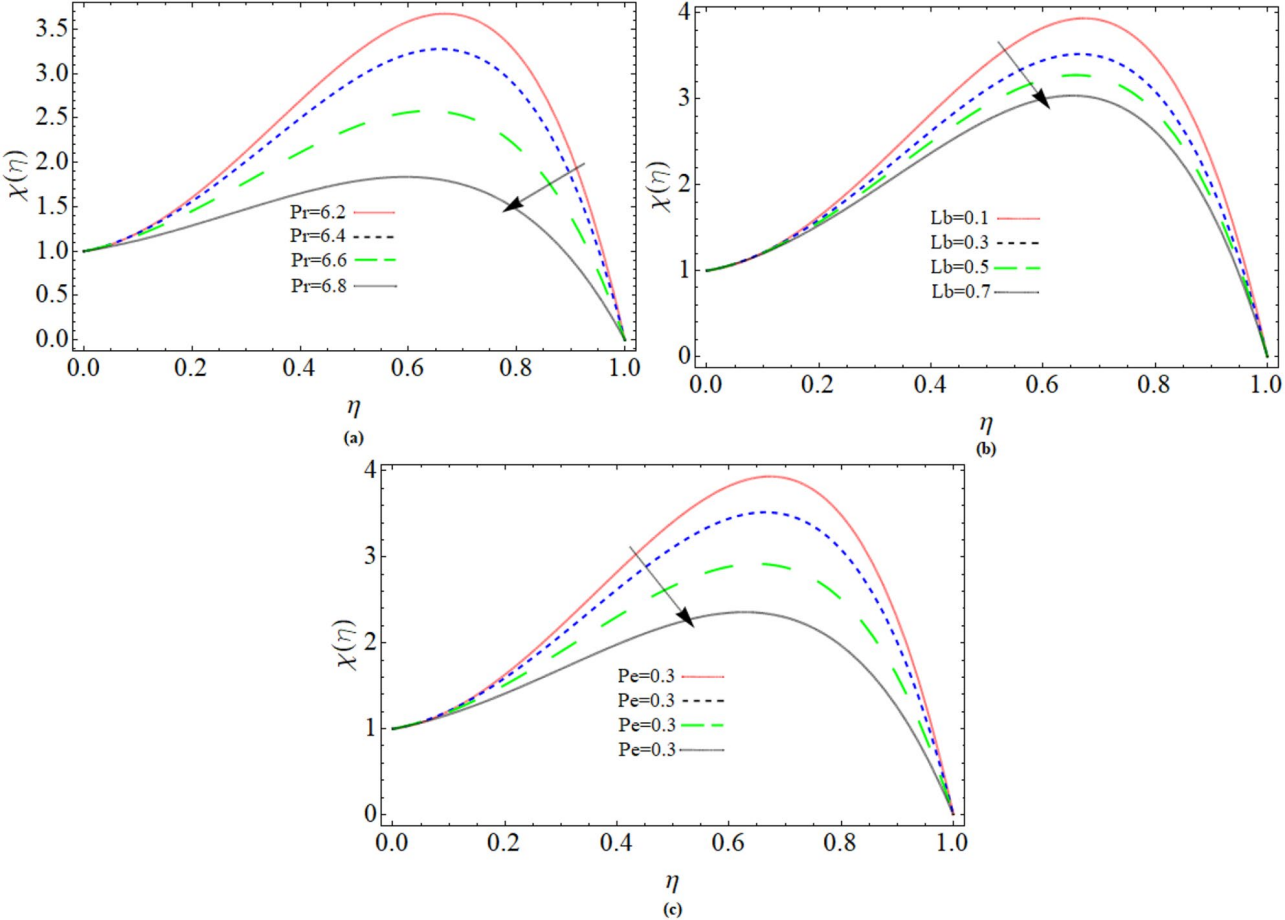
Prandtl number *Pr*, Bioconvection Lewis number *Lb* and Peclet number *Pe* effect on gyrotactic microorganism profiles $\chi \left( \eta \right)$.

Prandtl number *Pr*, *Lb* and *Pe* effect on gyrotactic microorganism profiles $\chi \left( \eta \right)$ has been shown through Fig. 6a–c. The Gyrotactic Microorganism profile reduces versus the action of *Pr*, *Lb* and *Pe* respectively. With distinct Prandtl numbers, the thickness of the hydrodynamic boundary layer and the thickness of the thermal boundary layer are calculated physically. If *Pr* = 1, that means the thermal boundary layer's thickness is the same as the velocity boundary layer's thickness. As a result, it's the momentum-to-thermal-diffusivity ratio. That’ why, fluid temperature reduces with growing value of Prandtl number as demonstrated in Fig. 6a. Similar trend has been observed of Gyrotactic Microorganism profile versus Bioconvection Lewis number *Lb* and Peclet number in Fig. 6c,d. Table 1 revealed the comparison of PCM technique with the existing literature, while varying n and M. The rest of parameters were chosen zero. Table 2 illustrate the numerical outcomes for skin friction and Nusselt number versus several physical constraints. The temperature of the sheet surface rises when the magnetic field parameter is increased, as shown in Table 2.

**Table 1:** The comparative analysis for $- f^{\prime}\left( 0 \right).$

n	*M*	Chen^28^	Ferdows et al.^27^	Present work
0.4	0.0	1.27294	1.2732173	1.7232173
	0.5	1.81095	1.81174012	1.81174021
	1.0	2.28377	2.38406002	2.38407113
0.8	2.0	3.12650	3.12670459	3.12681321
	0.0	1.02919	1.02923546	1.02925732
	0.5	1.30790	1.30794967	1.30797561
	1.0	1.54411	1.54412572	1.54419823
	2.0	1.94532	1.94533236	1.92589745
1.0	0.0	1.0000	1.00000000

**Table 2:** Skin friction $C_{f} Re_{x}^{{1/\left( {n + 1} \right)}}$ and Nusselt number $Nu_{x} Re_{x}^{{ - 1/\left( {n + 1} \right)}}$ for various physical parameters.

n	*M*	$\Phi \left( 0 \right)$	$C_{f} Re_{x}^{{1/\left( {n + 1} \right)}}$	$Nu_{x} Re_{x}^{{ - 1/\left( {n + 1} \right)}}$
0.5	0.0	1.024605	− 1.680869	0.976181
	1.0	1.263663	− 2.472113	0.791512
	5.0	2.723158	− 3.881251	0.367333
1.0	0.0	0.867808	− 1.345121	1.152097
	1.0	0.980749	− 2.323703	1.019633
	5.0	1.444195	− 4.529539	0.962375
1.5	0.0	0.808309	− 1.164934	1.127858
	1.0	0.886658	− 2.189181	1.127858
	5.0	1.158166	− 4.836690	0.863408
1.8	0.0	0.782458	− 1.073400	1.278088
	1.0	0.847508	− 2.107885	1.180170
	5.0	1.052396	− 4.981860	1.950203

## Conclusion

The current research investigates the movement of an incompressible, steady power law nanoliquid containing gyrotactic microorganisms between two parallel plates with heat transmission. Only one plate moves in relation to another at a time. Nonlinear partial differential equations have been used to model the problem (PDEs). Which reduced form (ordinary differential equations) is solved through the Parametric Continuation Method (PCM). The results are related to the boundary value solver (bvp4c) approach for validation and accuracy purposes. The below are the objectives:

- The velocity profile shows positive response versus the rising values of Buoyancy convection parameters. While reduces with increasing effect of magnetic field, because magnetic impact generated Lorentz force, which reduces fluid velocity $f\left( \eta \right)$.
- The rising effect of magnetic Prandtl number and Prandtl number significantly reduces the fluid temperature.
- Brownian behavior is considered as the non-movement of fluid molecules over the plate's surface. Brownian motion induces heat by increasing the unspecific motion of liquid particles. As a result, the liquid temperature rises, as does the thickness of the thermal boundary layer.
- The concentration distribution declines with rising values of Lewis number *Le*. Because the rate of molecular diffusion reduces, and kinematic viscosity enhances with the effect of Lewis number.
- With Peclet number Pe and Bioconvection Lewis number, the density of motile microorganisms falls.

## Data Availability

The data that support the findings of this study are available from the corresponding author upon reasonable request.

## Symbols

$$U_{w}$$: Stretching velocity
$$g$$: Acceleration
$T_{\infty }$: Free stream temperature
$$\mathop{j}\limits^{\rightharpoonup} \times \mathop{B}\limits^{\rightharpoonup} $$: Lorenz force
$$\sigma_{e}$$: Electrical conductivity
$$B_{0}$$: Magnetic field
$c_{p}$: Specific heat capacity
$D_{n}$: Diffusivity of microorganism
*K*: Thermal diffusion ratio
$$\mu_{2}$$: Magnetic permeability
*Pe*: Peclet number
$\lambda_{1}$: Buoyancy convection coefficient due to temperature
$\lambda_{3}$: Buoyancy convection coefficient due to microorganism
$$C_{f}$$: Skin friction
$$Sh_{x}$$: Sherwood number
$$q_{w}$$: Heat flux
$q_{n}$: Motile microorganism flux
*A*: Matrix coefficient
$h^{*}$: Gap between two plates
$$T$$: Fluid temperature
$\beta$: Heat source/sink parameter
$$D_{T}$$: Thermophoresis coefficient
$$D_{B}$$: Brownian motion
*C*: Fluid concentration
$\tau$: Effective heat capacitance
$W_{c}$: Cell swimming speed
$$\rho$$: Density
$Pr_{m}$: Modified Prandtl number
*Le*: Lewis number
$\lambda_{2}$: Buoyancy convection coefficient due to concentration
*U, W*: Vector functions
$$Nu_{x}$$: Nusselt number
$Nn_{x}$: Density of motile microorganism
$$\tau_{w}$$: Shear stress
$$q_{m}$$: Mass flux
PCM: Parametric continuation method
